# Exploring the Physiological Link between Psoriasis and Mood Disorders

**DOI:** 10.1155/2015/409637

**Published:** 2015-10-15

**Authors:** Cody J. Connor, Vincent Liu, Jess G. Fiedorowicz

**Affiliations:** ^1^Department of Family Medicine, Broadlawns Medical Center, Des Moines, IA 50314, USA; ^2^Carver College of Medicine, University of Iowa, Iowa City, IA 52242, USA; ^3^Department of Dermatology, University of Iowa, Iowa City, IA 52242, USA; ^4^Department of Pathology, University of Iowa, Iowa City, IA 52242, USA; ^5^Department of Psychiatry, University of Iowa, Iowa City, IA 52242, USA; ^6^Department of Internal Medicine, University of Iowa, Iowa City, IA 52242, USA; ^7^Department of Epidemiology, College of Public Health, University of Iowa, Iowa City, IA 52242, USA

## Abstract

Psoriasis is a chronic, immune-mediated skin condition with a high rate of psychiatric comorbidity, which often goes unrecognized. Beyond the negative consequences of mood disorders like depression and anxiety on patient quality of life, evidence suggests that these conditions can worsen the severity of psoriatic disease. The mechanisms behind this relationship are not entirely understood, but inflammation seems to be a key feature linking psoriasis with mood disorders, and physiologic modulators of this inflammation, including the hypothalamic-pituitary-adrenal axis and sympathetic nervous system, demonstrate changes with psychopathology that may be contributory. Cyclical disruptions in the secretion of the sleep hormone, melatonin, are also observed in both depression and psoriasis, and with well-recognized anti-inflammatory and antioxidant activity, this aberration may represent a shared contributor to both conditions as well as common comorbidities like diabetes and cardiovascular disease. While understanding the complexities of the biological mechanisms at play will be key in optimizing the management of patients with comorbid psoriasis and depression/anxiety, one thing is certain: recognition of psychiatric comorbidity is an imperative first step in effectively treating these patients as a whole. Evidence that improvement in mood decreases psoriasis severity underscores how psychological awareness can be critical to clinicians in their practice.

## 1. Introduction

Psoriasis vulgaris is a chronic, autoimmune, inflammatory skin disorder phenotypically characterized by clearly demarcated, salmon-colored plaques surfaced by micaceous, silvery scale. It is considered to be immune-mediated, arising with increase and activation of cutaneous T cells and dendritic cells [1]. Following migration to the skin, these immune cells release proinflammatory cytokines, such as interleukin-1 (IL-1), IL-6, and tumor necrosis factor *α* (TNF-*α*), which promote inflammation and keratinocyte proliferation. These keratinocytes accumulate without shedding, resulting in the distinctive patches of thickened, scaly skin. Although often underappreciated, psoriasis can result in considerable disability to afflicted patients, and some estimate that the burden imparted is comparable to other major diseases like chronic heart failure, chronic obstructive pulmonary disease, and cancer [2].

Psychiatric comorbidity is estimated to affect over 30% of patients with dermatologic disease [3]. The prevalence of depression and anxiety in those with psoriasis, specifically, is significantly higher than that observed in the general population [4], and in comparison to many other dermatologic conditions, psychiatric morbidity is higher and quality of life scores are lower [5–9]. One might attribute the association between psoriasis and mood disorders as simply resulting from the related embarrassment, shame, and social anxiety incurred from the physical manifestations of disease; however, the fact that the prevalence of mood symptoms in psoriasis is higher than that observed with many other disfiguring skin disorders [5–9] begs for investigation of common or overlapping mechanisms linking both conditions.

## 2. The Role of Inflammation

There is a growing body of literature supporting some link between major depressive disorder and inflammation [10]. Elevations in proinflammatory cytokines like prostaglandin E2 (PGE2), C-reactive protein (CRP), TNF-*α*, IL-1*β*, IL-2, and IL-6 have been reported in major depressive disorder and, at times, have shown a dose-response with severity of depression [10, 11]. These findings, however, cannot distinguish whether the depression is causing the inflammation or vice versa. In exploring this question of temporality, there is evidence and biological plausibility for both directions.

### 2.1. Depression Causing Inflammation

Could depressive states induce elevations in the inflammatory cytokines previously mentioned? A randomized controlled trial revealed that antidepressant treatment with the selective serotonin reuptake inhibitor (SSRI), sertraline, significantly decreased not only depression scores, but also baseline measurements of CRP and IL-6 in depressed subjects [12]. A large meta-analysis also demonstrated that psychological stress elevates inflammatory markers like CRP, TNF-*α*, IL-1*β*, and IL-6 [13]. The net effect is an acute increase in the proinflammatory potential of the circulating immune system, and in the setting of psoriasis, this could mean exacerbation of disease. In fact, 40–80% of cases of psoriasis onset or exacerbation have been attributed to psychosocial factors, revealing a significant role for mood in modulating disease [14]. While there has been limited empirical study of those with cooccurring depression and psoriasis, a case report describes a young adult woman with schizoaffective disorder who experienced consistent worsening of psoriasis during periods of increased depression and suicidality with improvement during periods of normal mood [15]. Exacerbation of psoriasis with worsening depression and anxiety is common, and a variety of physiological pathways may be involved in mediating this relationship.

One particularly plausible pathway involves abnormal activation of the hypothalamic-pituitary-adrenal (HPA) axis. Various studies have identified chronically elevated baseline levels of corticotropin releasing hormone (CRH), adrenocorticotropic hormone (ACTH), and cortisol in depressed individuals [16]. This HPA-axis hyperactivity may result from changes in the number and function of cortisol receptors functioning in negative feedback. The effects of cortisol are mediated by two different types of intracellular receptors, the mineralocorticoid receptor (MR, type I) and the glucocorticoid receptor (GR, type II) [17]. MRs have a much higher affinity (10x) for endogenous glucocorticoids like cortisol. GRs, on the other hand, bind strongly to synthetic steroids like dexamethasone. Given these profiles, MRs are believed to primarily moderate cortisol's effects and its feedback on the HPA axis when cortisol levels are low. During periods of acute stress, cortisol levels may increase 100-fold, effectively saturating the MRs and leaving the GRs as the main regulators of glucocorticoid action and HPA activity [18].

Decreases in the number or function of GRs may thus result in decreased negative feedback by cortisol, leading to the HPA hyperactivity demonstrated in depressed individuals. This mechanism is supported by reports of cortisol nonsuppression following administration of dexamethasone—which, recall, normally has strong GR affinity—in subjects with major depression: an effect that disappears with clinical recovery from depression [16]. Hypercortisolemia appears to produce negative effects on mood and cognition. These effects may be mediated mainly through MRs, or possibly through GRs that have not lost their functional capacity as the ones in the hypothalamus, which participate in HPA autoregulation.

### 2.2. HPA Axis and Sympathetic Nervous System Effects on Skin and Inflammation

HPA hyperactivity appears not only to influence mood, but also to effect changes in the skin. CRH has been shown to stimulate local cutaneous production of cytokines like IL-6 and IL-11 [19], and it also amplifies cytokine-stimulated keratinocyte expression of the immune trafficking adhesion molecules, hCAM and ICAM-1, and the major histocompatibility complex II receptor, HLA-DR [20]. In addition, CRH activates the proinflammatory protein complex NF-*κ*B (nuclear factor kappa-light-chain-enhancer of activated B cells), which is present in almost all cell types and modulates DNA transcription and immune reaction in response to stimuli like stress, UV light, free radicals, and bacterial/viral infection [21]. Through all of these changes, CRH effectively shifts keratinocytes into an immunoactive state [20] and thus may contribute to inflammatory skin conditions, such as psoriasis. In fact, biopsies of psoriatic lesions show significantly increased expression of CRH compared to normal skin, suggesting a possible role of CRH in the underlying pathophysiologic mechanism of the disease [22].

Evidence suggests that peripheral CRH is part of an HPA-like system that functions locally within the skin and hair follicles [23, 24]. Whether produced locally or delivered by peripheral nerves, CRH may be a key component of the “brain-skin axis,” mediating interactions between the central and peripheral stress response pathways [24]. Downstream HPA products, like cortisol, are also known to act peripherally within this cutaneous system. Administration of systemic glucocorticoids in rodents has been shown to cause adverse changes in the skin, including disruption of epidermal cell proliferation [25] and barrier homeostasis [26]. These cutaneous effects are blocked by simultaneous administration of the steroid hormone receptor antagonist, RU-486, effectively identifying glucocorticoids as the mediators through which stress may disturb cutaneous quiescence [26].

The way in which the HPA axis is affected appears to differ amongst psoriasis patients depending on whether they suffer from depression or anxiety. Those with depression have chronically elevated levels of CRH, ACTH, and cortisol, while those with stress-responsive psoriasis (psoriasis that worsens during periods of high stress) have been shown to have a blunted HPA-stress response, with less cortisol released into circulation following acute stress [27]. Even so, it may be that depression and anxiety both worsen the severity of psoriasis, only through separate mechanisms. In those with stress-responsive psoriasis, acute anxiety may cause the production of inflammatory cytokines without the appropriate release of anti-inflammatory cortisol to mitigate the cutaneous response [27].

One study found that chronic psychological stress prolongs epidermal permeability barrier recovery following intentional disruption by repeated stripping with cellophane tape, and the extent of this prolongation was directly correlated with the patients' measured stress levels [28]. This demonstrates the influence that stress can have on the skin and is of particular importance in psoriasis, which has been associated with abnormalities in epidermal barrier function that are directly related to severity of the psoriatic lesions [29]. It is interesting to consider that this observed prolongation of barrier recovery may be due to an overall effect of anxiety on healing in general. It is true that chronic psychological stress has been observed to delay wound healing both in rats [30] and in humans [31, 32]. The consequence of this effect may be particularly meaningful in psoriasis, where the Koebner phenomenon (lesions forming at sites of trauma) is a commonly observed source of worsening disease. By disrupting normal barrier homeostasis and slowing wound healing, anxiety may sensitize the psoriatic patient to the formation of trauma-induced plaques and/or cause delay in resolution of such lesions [28].

Beyond HPA axis contributions, there may also be a role for autonomic modulation via the sympathetic nervous system (SNS). Anxiety, in particular, has been extensively correlated with sympathetic activation and vagal inhibition [33]. Likewise, those with depression demonstrate increased sympathetic tone, with elevated muscle sympathetic nervous system activity (MSNA) both at rest and during mental stress: an effect that is more pronounced as depression scores worsen and is reduced by treatment with sertraline [34]. Plasma norepinephrine—another measure of SNS activity—is also higher in those with major depression, and this normalizes after treatment with antidepressants like SSRIs [35] and desipramine [36].

The SNS innervates both primary and secondary lymphoid organs and is known to promote inflammation [37]. Studies have revealed sympathetic-mediated upregulation of proinflammatory cytokines like IL-6 and IL-1*β* [38], as well as increased mobilization and release of bone-marrow-derived hematopoietic stem and progenitor cells into circulation [39]. In particular, norepinephrine appears to be the main effector in this sympathetic enhancement of immune activity [40]. Saint-Mezard et al. demonstrated that psychological stress experienced prior to immunization afforded an “adjuvant” effect upon the resulting inflammatory reaction by augmenting dendritic cell response and consequently increasing primary and memory T-cell activation [40]. Through selective depletion of NE, they were able to further determine that this effect was mediated by NE and not stress-induced glucocorticoid release, as NE depletion effectively normalized dendritic cell migratory activity and the ensuing hypersensitivity reaction. Psoriatic patients have been found to secrete excess NE in response to stress as compared to control patients [41, 42]: a finding that might help explain the apparent stress responsiveness of the condition and the characteristic cytokine milieu of elevated TNF-*α* and IFN with increased trafficking of lymphocytes and dendritic cells to the skin.

Of the receptor subtypes involved in the sympathetic stress response, the alpha adrenergic receptors (*α*-ARs) have been repeatedly revealed as the mediators bringing about increased cytokine production and proinflammatory changes [43]. Norepinephrine released in response to stress activates *α*-ARs present in macrophages and dendritic cells, subsequently leading to increased release of TNF-*α* and suppression of anti-inflammatory IL-10 [43]. Of course, such an effect would reasonably lead to exacerbation of an inflammatory condition already brewing in a patient, such as psoriasis. In contrast, activation of *β*_2_-ARs in these same immune cells elicits a suppression of TNF-*α* release and promotes IL-10 production, and in T cells, *β*_2_ activation inhibits production of IL-2, which is needed for lymphocyte proliferation required to mount an ample immune response [43].

Given the opposing actions of these two AR subclasses, the particular immunologic stress reaction imparted in an individual is dependent upon the differential expression of *α*- and *β*-ARs within that subject and the ultimate balance of their effects as a whole. Lubahn et al. discovered that, in the chronic inflammatory disease, rheumatoid arthritis, treatment with *β*_2_-agonists did not significantly elicit a change in cytokine profiles or immune function while administration of an *α*-antagonist did produce such an effect [43]. They concluded that chronic inflammatory disease alters the ability of the SNS to regulate immunoreactivity through ordinary *β*_2_-AR signal transduction, effectively shifting toward *α*-ARs as the primary receptor class regulating immune function in such conditions. As psoriasis, like rheumatoid arthritis, is a disease of chronic inflammation, it is not unreasonable to imagine that a similar mechanism may explain the stress-responsiveness of the disease. In fact, use of *β*-blocker medication has been well-reported in the literature to cause onset of psoriasis and exacerbation of already present disease [44, 45], providing evidence that this may indeed be the case.

Presumably by further diminishing the anti-inflammatory effects of *β*-AR stimulation, *β*-blockers appear to augment the inflammatory actions of *α*-ARs, causing worsening of disease. While the downstream effects on TNF-*α* and IL-10 have already been indicated as key components of this pathway, there also appears to be a role for fibroblast growth factor 10 (FGF10) in determining the influence of the SNS on psoriasis. Studies have found that FGF10 causes keratinocyte proliferation as seen in psoriatic disease [46, 47], and immunostaining has discovered increased expression of FGF10 in psoriatic lesions, positively correlating with the amount of T-cell infiltrate detected [46]. In further exploring the influence of FGF10 in psoriasis, Yao et al. found that neutralization of FGF10 with a monoclonal antibody significantly improved *β*-blocker-induced psoriasis in guinea pigs, in terms of both epidermal thickness and monocyte infiltrate [47]. This reaffirmed that FGF10 exerts an effect on psoriasis severity and that disruption of normal *β*-AR activity—as observed in chronic diseases like rheumatoid arthritis or psoriasis—can allow for this effect to rise above the normal anti-inflammatory actions, otherwise keeping it in check. The overarching conclusion conveyed by these studies is that SNS activity, triggered by acute stress, generates NE that disproportionately activates proinflammatory *α*-ARs in the setting of chronic disease, and in psoriasis, this can lead to worsening of disease by means of various chemical messengers including TNF-*α* and FGF10.

Some have theorized that the adjuvant-like effect of acute stress on immunoreactivity is an evolutionary adaptation, the purpose of which is to prepare the body to fight off infection following injury [48, 49]. The interesting thing about mankind is that each individual interprets their environment through their own eyes, affected by their personal set of experiences, wisdom, coping mechanisms, fears, and expectations. It is the subjective interpretation of a stressor that dictates one's reaction and subsequent immune response, suggesting that inflammatory responses should be less likely in those that “go with the flow” or do not become as anxious in the face of stressors as others [48]. Research has shown that this is indeed the case. Carroll et al. found that task related increases in anxiety and anger are directly correlated with serum levels of inflammatory IL-6, demonstrating that affective responses are instrumental in determining the degree of subsequent immune reactivity in an individual [50]. Others have reproduced this same result and, in addition, discovered that perceived social support diminishes the relationship between subjective stress experience and IL-6 increase [51].

This discussion is highly pertinent when considering the way that depression might influence psoriasis, as those with depression suffer from cognitive distortions that alter the ways in which they interpret their environment [52, 53]. This includes decreases in perceived degree of social support among depressed subjects: an effect that improves with effective treatment and resolution of depressive symptoms [53]. While the increased stress immune responsiveness of patients with low social support might be an evolutionarily advantageous adaptation, it becomes detrimental when the immune system is already active and directed in a pathological process, such as psoriasis. Under such circumstances, acute stress only amplifies the present disease process and worsens disease severity rather than enhancing infection defenses as evolutionary pressures intended.

Beyond the effects of acute stress, chronic stress is likely to be a predominant issue for patients with depression/anxiety. How do the consequences of chronic stress differ, and how might they alternatively impact the course of inflammatory disease? There is ample evidence that chronic stress can result in immunosuppression or immune dysregulation [49], with the exact effect determined by a number of individual factors. As similarly discussed in regard to depression, chronic stress results in lasting HPA axis hyperactivity with elevated baseline levels of ACTH, CRH, and cortisol. Physiologic levels of glucocorticoids, unlike pharmacologic levels, have been found to actually enhance immune responses by increasing T-cell responsiveness to IL-2, promoting production of cytokines like IL-1 and IL-6, and supporting biological sensitivity to cytokines like granulocyte colony-stimulating factor, granulocyte macrophage colony-stimulating factor, and IFN-*γ* [49]. In addition, HPA axis response to acute stress is blunted, meaning anti-inflammatory cortisol does not appropriately elevate, and the normal diurnal cycle of cortisol secretion in the body is also disrupted [54]. These changes appear to correlate with the harmful effects of chronic stress [49]—including decreased wound healing, as previously reviewed, and promotion of inflammation through increased production of inflammatory cytokines and a shift toward type 2 cytokine-mediated immune responses [49, 55]—and are thought to be the primary means by which stress induces or exacerbates autoimmune and allergic diseases.

With all considered, depression and anxiety likely contribute to the worsening of inflammatory disorders, like psoriasis, via both HPA axis and SNS hyperactivity. Those with mood disorders demonstrate baseline disruptions in these physiologic systems that contribute to ongoing immunopathology. Additionally, these populations show increased responsiveness to acute stressors, which further alters immune function and can acutely worsen chronic disorders of inflammation and autoimmunity.

### 2.3. Inflammation Causing Depression

While the discussion thus far has centered on the ways in which depressive or anxious states appear to increase inflammation, induction of an inflammatory state, as in psoriasis, can also precipitate changes in mood, including depressive symptoms. One study found that healthy volunteers vaccinated for* Salmonella typhi* subsequently displayed increases in inflammatory markers, like IL-6, TNF-*α*, and IL-1Ra, as well as depressive symptoms without signs of physical sickness [56]. Other researchers have witnessed the induction of “sickness behavior” in rats following injection of IL-1 and lipopolysaccharide (LPS). This “sickness behavior” included decreases in energy, sleep, appetite, interest exploring, and sexual activity: a presentation reminiscent of major depression [57]. Furthermore, baseline CRP and IL-6 measurements have been observed to effectively predict future depression at 12-year follow-up, while baseline symptoms of depression were not predictive of future inflammatory markers [58]. A randomized control trial using the TNF-*α* inhibitor, etanercept, revealed that treatment of inflammation in the setting of psoriasis results in measured improvements in fatigue and depressive symptoms [59]. While decreases in fatigue were correlated with decreased joint pain, improvements in depressive symptoms were less correlated with psoriasis and arthritis severity, suggesting the presence of an alternative mechanism to explain this particular effect: potentially the physiologic changes associated with decreased systemic inflammation as opposed to the psychological effects of decreased psoriasis severity. All of these studies demonstrate the potential for inflammation, and thus psoriasis, to induce depression, and one possible mechanism to explain this effect involves alterations in the metabolism of serotonin.

Cytokines, more specifically IL-2 and IFN-*α*, have been shown to directly increase the enzymatic activity of indoleamine 2,3-dioxygenase (IDO), which increases the conversion of tryptophan to kynurenine [60]. Shunting of tryptophan toward kynurenine consequently decreases the production of serotonin from tryptophan, resulting in a functional serotonin deficit that might produce depressive symptoms. Compounding this effect, tryptophan metabolites, including kynurenine, have been found to independently induce depressive symptoms [61]. Besides activation via inflammatory cytokines, this pathway is also activated following induction of tryptophan 2,3-dioxygenase (TDO) by glucocorticoids: another potential explanation for how the hypercortisolemia seen in depression may contribute to symptomatology [61]. Inflammatory cytokines like IL-6 have also been found to increase the breakdown of serotonin [62]. Thus, inflammation may affect mood by simultaneously decreasing production and increasing degradation of serotonin.

## 3. The Role of Melatonin

Inflammatory cytokines are not the only biomarkers linking depression and psoriasis. Depression is associated with disruptions in the secretion of melatonin [63], which physiologically follows a circadian rhythm with elevated levels at night, usually peaking in the early morning between 2 and 4 a.m. [64]. Melatonin is well known for regulating the daily sleep cycle, but it also modulates immune function. By reducing levels of TNF-*α*, IL-6, and IL-8, melatonin can theoretically attenuate the severity of inflammatory disorders [65, 66]. Melatonin dysregulation has been observed in other inflammatory conditions, as well, including sarcoidosis and, indeed, psoriasis vulgaris [67, 68]. Such disruptions in cyclic melatonin secretion could contribute to the inflammation observed in these disease states. Nighttime melatonin levels have been recorded to be significantly lower in patients with psoriasis than in healthy controls [64]. Decreased melatonin levels could contribute to the previously mentioned Koebner phenomenon responsible for exacerbating psoriasis, as absence of melatonin through pinealectomy has been shown to delay wound healing in rats, while melatonin replacement in these rats effectively eradicated this effect [69].

In those with depression, decreased melatonin levels and a dysfunctional circadian rhythm may disinhibit the release of melanocyte stimulating hormone (MSH), which has been linked to seborrhea in rats and may play a role in psoriasis [15, 70]. In addition, hypersecretion of CRH results in the production of ACTH from its precursor molecule, proopiomelanocortin (POMC). As a byproduct of ACTH synthesis from POMC, MSH levels would be expected to increase under such circumstances: an effect seen in Cushing's disease where the overproduction of ACTH can, through associated elevations in MSH, physically manifest itself as generalized hyperpigmentation [71].

Thus, MSH may be elevated in depression due to both low melatonin levels and hypersecretion of CRH, and this could contribute to the presentation of patients with psoriasis. Interestingly, studies have implicated MSH as a contributor to depressive symptoms [72, 73], and administration of an MSH inhibitor has been observed to significantly improve depressive symptoms [74]. Further research is certainly needed to better characterize the role that cyclic changes in melatonin play in depression and psoriasis, and the mechanism by which this role is achieved.

Low melatonin may also play a role in the morbidity of psoriasis. A prospective cohort study with 14,128 subjects demonstrated a significantly increased risk for developing diabetes mellitus type II (DMII) in psoriasis patients versus the comparison group, and this risk was directly related to the severity of the psoriasis [75]. Interestingly, melatonin has been shown to participate in the regulation of blood glucose levels via melatonin receptors within the pancreatic beta cells and insulin receptors in the pituitary gland [76]. The interrelationship has been demonstrated in DMII, where increased levels of insulin are accompanied by decreased levels of melatonin, decreased expression of pineal insulin receptors, and increased pancreatic expression of melatonin receptors [76]. In fact, there is evidence for a circadian rhythm of insulin secretion, which demonstrates an inverse relationship between insulin and melatonin levels [77]. The link between melatonin and diabetes has not yet been fully elucidated, but DMII is associated with decreased levels of melatonin, and recent genome-wide association studies have demonstrated that single nucleotide polymorphisms of the MT2 melatonin receptor locus significantly increase the risk for developing DMII [78–80]. Others have demonstrated that pinealectomy in rats results in insulin resistance and decreased expression of GLUT 4 receptors, with return to control values following melatonin replacement [81].

The mechanism behind the apparent protective effects of melatonin is not established, but available evidence offers some clues. Some studies have found that melatonin might sensitize pancreatic beta cells [82] or even induce regeneration and proliferation of beta cells that have been destroyed in streptozotocin-induced type I diabetes [83–85]. Its activity as a free radical scavenger [86, 87] provides protection to pancreatic beta cells which, themselves, have low antioxidative capacity and are thus susceptible to oxidative damage [88]. Several publications support the notion that diabetes creates a state of increased free radical production [89–91], so decreased melatonin may represent a possible contributing factor as well as a target for intervention via melatonin supplementation. In fact, melatonin has been shown to decrease insulin resistance in rats with DMII, reportedly by improving lipid metabolism [92].

Insulin resistance has also been associated with major depression, a condition which, as discussed, also demonstrates abnormalities in melatonin secretion and is a common comorbidity of psoriasis. Might abnormalities in melatonin be the link between psoriasis, depression, and diabetes? This conclusion cannot be drawn by the simple associations observed, but melatonin levels naturally decline with age, and this may contribute to the incidence of diabetes as well as the other inflammatory conditions mentioned, including psoriasis. Both later-onset (type II) psoriasis [93] and DMII develop with age in those predisposed, and this may partially result from a drop in melatonin levels beyond a threshold necessary for counteracting the disease process. This is similar to the suggestion by some researchers that the age-related decline of melatonin may be contributory to the increased incidence of breast cancer in older women [94]. Of course, melatonin is not the only neuroendocrine factor that fluctuates with age, and it is likely that other such factors are also at play in modulating the relationship between psoriasis, diabetes, and inflammation.

In addition to depression and diabetes, psoriasis is associated with a variety of conditions in which chronic inflammation plays a pathophysiological role, including myocardial infarction, hypertension, stroke, metabolic syndrome, and cardiovascular mortality [75]. Melatonin may more directly affect these morbidities, and psoriasis itself, via its well-supported anti-inflammatory properties. For example, melatonin administration has been shown in multiple reports to decrease blood pressure in untreated, hypertensive men and even in adolescents with type I diabetes [95–97]. In addition, it has also been observed to protect against endothelial dysfunction [98] and ischemic heart failure [99], further illustrating the potentially widespread clinical applications of melatonin, particularly in the at-risk psoriatic population.

Clinical practice may already hint at the significance of melatonin in both depression and psoriasis. Phototherapy is a common, and effective, treatment for depression, and it is also a current treatment for psoriatic lesions. The mechanism underlying its usefulness in psoriasis is not entirely known, but the current conceptual model implicates inhibition of keratinocyte proliferation, and more importantly, immunomodulation via altered cytokine composition and cytokine receptor expression decreased adhesion molecules necessary for immune cell trafficking, altered types and functions of antigen presenting cells, and induction of lymphocyte apoptosis [100–103]. Considering that sunlight serves as the main zeitgeber in setting the circadian rhythm of melatonin secretion, it is possible that normalization of melatonin derangement may be another benefit of phototherapy, as long as light exposure includes the retina. Melatonin may be a candidate for study as a treatment option for comorbid depression and psoriasis, given its potential to address the aberrations in cyclical melatonin secretion observed in both conditions. In fact, the melatonin receptor agonist, agomelatine, has already shown effectiveness in treating depressive symptoms, further demonstrating the influence this hormone has in mood disorders.

## 4. Integrated Treatment

Not only is managing depression important for enhancing the patient's quality of life, but it may also be helpful in improving his/her psoriasis. According to one study, three negative schemas—vulnerability to harm, defectiveness, and social isolation—predict anxiety and depression in psoriasis patients [104]. The apparent role of such cognitive distortions may support the use of cognitive behavioral therapy (CBT) in this population. A case-controlled study [105] involving 40 patients with psoriasis receiving adjunctive group CBT revealed greater reduction in depression and anxiety scores and nearly double the reductions in self-reported disability and life stress scores compared with the control group. Perhaps more interestingly, the treatment group also experienced a significant reduction in Psoriasis Area Severity Index (PASI) scores at 6 weeks and 6 months' follow-up, with 64% of patients achieving 75% psoriasis clearance at 6-month follow-up compared with 23% of the control group.

While SSRIs are often the first-line treatment for depression, fluoxetine has been observed to exacerbate psoriasis in some patients [106, 107]. Lithium, a common treatment for bipolar disorder, has also been reported to worsen psoriasis and even induce psoriasiform lesions in those without psoriasis [108, 109]. In both cases, standard psoriasis treatments are ineffective, with resolution ensuing only after lithium discontinuation [109]. To avoid aggravation of comorbid psoriasis in these sensitive populations, CBT might be an attractive alternative treatment to psychoactive medication, with a possible additional role for adjunctive melatonin supplementation.

## 5. Conclusion

**Figure 1:**
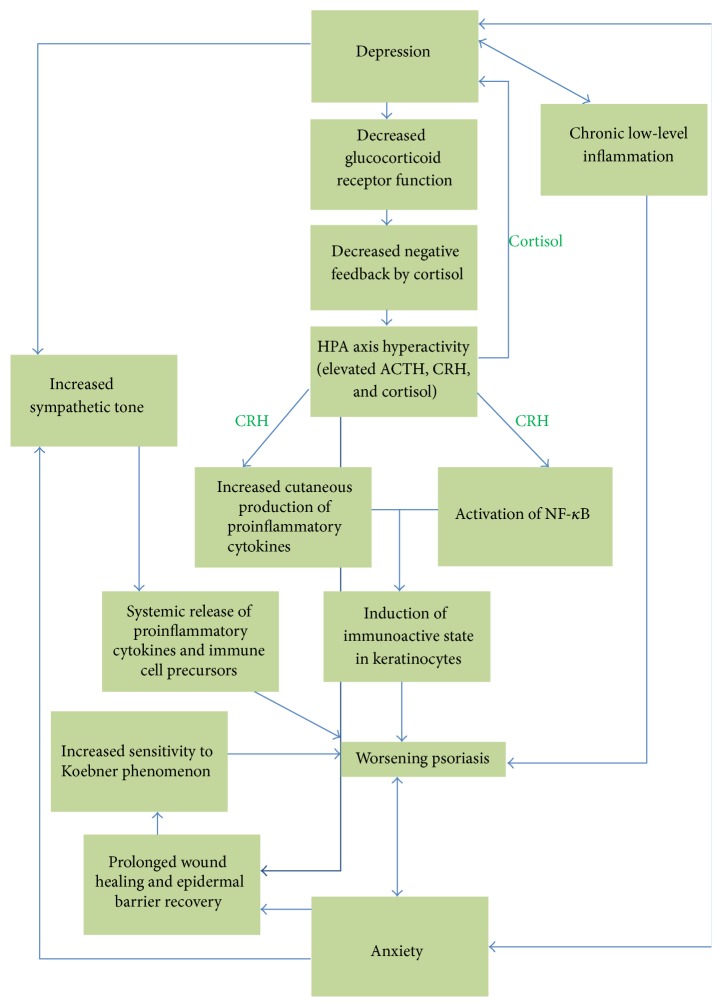
Overview: psoriasis and mood disorders. Depression and anxiety interact with psoriasis through associations with HPA axis hyperactivity, sympathetic hyperactivity, chronic inflammation, and delayed wound healing.

Overall, psoriasis vulgaris and mood disorders are highly associated, and despite frequent underdiagnosis, this combination of comorbidities causes patients much disability. Interestingly, fluctuations in mood appear to affect the severity of psoriasis (Figure 1) and, thus, may serve as a potential target for intervention [15, 110]. By ensuring that patients are properly screened and referred when depression is suspected, treatment can begin, morbidity can be prevented, and the psoriasis itself may improve in kind. The overlapping inflammatory cascades in both conditions could also represent a potentially important point of intervention, as addressing this could theoretically have a synergistic effect on improving psoriasis by mitigating both the basal inflammatory state and the depression and anxiety that cause exacerbations. In addition, it is intriguing to note the potential that melatonin offers in modulating various associations with psoriasis and mood disorders, including HPA axis abnormalities, epidermal barrier function deficits, diabetes, and the cardiovascular comorbidities associated with the chronic inflammation characteristic of these conditions.
